# Revisiting the Influence of Young White Wine Composition on Ester Evolution

**DOI:** 10.1021/acs.jafc.6c04425

**Published:** 2026-06-18

**Authors:** Diana A. Martin-Rojas, Tatjana Radovanović Vukajlović, Martin Šala, Vid Šelih, Valentina Obradović, Josip Mesić, Adrian Hermes, Melita Sternad Lemut, Guillaume Antalick

**Affiliations:** † Wine Research Centre, University of Nova Gorica, Lanthieri Palace, Glavni trg 8, 5271 Vipava, Slovenia; ‡ National Institute of Chemistry, Hajdrihova 19, 1000 Ljubljana, Slovenia; § Faculty of Tourism and Rural Development Požega, Josip Juraj Strossmayer University of Osijek, Vukovarska 17, 34000 Požega, Croatia; ∥ Centre for Information Technologies and Applied Mathematics, University of Nova Gorica, Lanthieri Palace, Glavni trg 8, SI-5271 Vipava, Slovenia

**Keywords:** ester hydrolysis, metals, matrix effect, storage, multivariate analysis

## Abstract

The evolution of volatile esters is a key determinant of the sensory profile of young white wines during the storage. This study evaluates the stability of 17 esters in 32 commercial wines under forced aging conditions. Although ester hydrolysis is typically associated with initial ester concentrations and pH conditions during storage, the results show that the wine matrix plays a major role in modulating reaction rates. Multivariate analysis identified metals, especially Fe and Mn, as significant drivers of ester degradation, suggesting a catalytic effect. In contrast, K and Mg showed an influence comparable to that of the pH. Overall metal composition and pH proved to be better predictors of shelf life than initial ester concentrations. These findings challenge traditional assumptions about ester equilibrium and highlight the importance of managing the metal content to preserve freshness and fruity aromas in young white wines over time.

## Graphical abstract

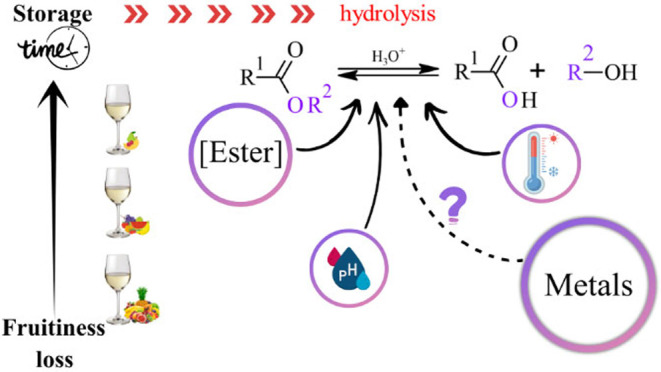

## 1 Introduction

Volatile esters play a significant role in the composition of wine’s aroma, imparting fruity notes, particularly important for young white wine quality. According to their synthesis pathway, they can be classified in three main groups: Ethyl Esters of Fatty Acids (EEFA), a product of straight chain fatty acids originating from yeast lipid metabolism and ethanol; Higher Alcohol Acetates (HAA), formed from higher alcohols via yeast nitrogen metabolism and acetyl CoA, and Ethyl Esters of Branched Acids (EEBA) synthesized from branched acids deriving from the same pathway as higher alcohols and ethanol. The conditions of alcoholic fermentation for white wines particularly favor EEFA and HAA production, making these groups the most representative in young white wines.^1^ These groups of esters are present in wines in the order of μg/L to mg/L, with ethyl hexanoate, ethyl octanoate, and isoamyl acetate displaying the highest concentrations in young white wines.^2^

During white wine storage, the evolution of esters follows a reversible kinetic reaction of hydrolysis and esterification (Scheme 1). The relative proportions of organic acids, alcohols, and ester forms will gradually tend toward equilibrium^3^ (the “Law of Mass Action”). After undergoing enzymatic accumulation during alcoholic fermentation, EEFA and HAA are usually in excess of their equilibrium concentrations and may hydrolyze in the period of aging, causing a loss of fruity aromas. On the other hand, branched organic acids, usually present in excess after alcoholic fermentation, will undergo esterification leading to the increase of EEBA concentration.^4^ Theoretically, the level of hydrolysis and esterification depends on the equilibrium between esters, organic acids and alcohols. Nevertheless, there is a consensus in the literature that pH, temperature, and postfermentation EEFA and HAA concentrations are the main parameters impacting hydrolysis dynamic.^2–4^ For instance, Ramey and Ough demonstrated that EEFA hydrolysis did not depend on ethanol concentration in wine conditions. Experiments performed by Makhotkina and Kilmartin in young Sauvignon Blanc wines also suggested the lack of relationship between HAA hydrolysis dynamics and corresponding levels of higher alcohols.

**Scheme 1:**
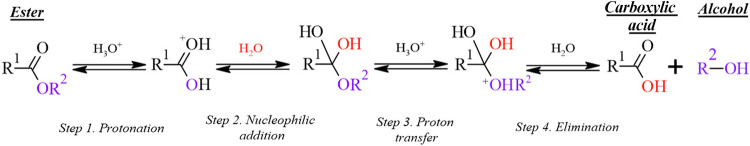
Reaction Mechanism of the Hydrolysis of Esters

In fact, the hydrolysis of esters in the wine matrix follows a pseudo-first-order rate law with respect to ester concentration. In this reaction, the protonation of the ester carbonyl function promotes its nucleophilic attack from water (eq 1). Despite the complexity of the wine matrix, the main required conditions for this reaction, such as an acidic medium and excess of water, are present.^3^ According to the differential form of chemical reaction rate (*r*) expressed in eq 1, high concentrations of esters following alcoholic fermentation favor an increase in the rate of hydrolysis, as the reaction rate is directly proportional to the ester concentration. The hydrolysis rate is strongly influenced by ester structure. Acetate esters of higher alcohols generally hydrolyze more rapidly than ethyl esters of fatty acids, reflecting their higher rate constants. Similarly, among ethyl esters, those with longer carbon chains are more susceptible to hydrolysis than short-chain analogues, consistent with their larger rate constants.^3,4^

1 $$hydrolysis\;rate\;\left(r\right)=\frac{-dc\left[esters\right]}{dt}=k_{obsd}c\left[esters\right]$$

Nevertheless, the evolution of esters during storage has been documented differently according to the type of wine (red or white). In general, ethyl decanoate and ethyl dodecanoate tend to decrease in both wine types, whereas shorter carbon chain EEFA remain relatively stable during aging. In contrast, HAA ester concentrations decrease considerably in white wines compared to red wines.^2^ Conversely, EEBA are initially present at low concentrations in young wines but progressively increase during aging.^2,5^

Ester hydrolysis can be affected by several factors, with pH and temperature being the most established influencing parameters. Marais^6^ demonstrated in a wine matrix that just slight changes in pH and temperature can promote the reaction of hydrolysis. The influence of other factors, such as sulfur dioxide (SO_2_) and other antioxidants on ester hydrolysis has been studied, suggesting that hydrolysis is less effective when antioxidants are present in the wine matrix. These antioxidants include *N*-acetyl-cysteine, caffeic acid, glutathione, and the use of inactive dry yeast.^7–9^ Nevertheless, the impact of antioxidants on ester evolution has not been clearly connected to oxygen. Patrianakou and Roussis^10^ showed that exposing wines to air during bottle aging could affect the final concentration of esters compared to wines treated with nitrogen, suggesting that an oxidative environment affects ester hydrolysis. In contrast, Radovanović Vukajlović et al.^11^ found that the exposition of a white wine to air during aging did not affect ester hydrolysis, excluding long carbon chain EEFA, which were more sensitive to the presence of oxygen. However, oxygen cannot interact directly with esters as it generally activates some intermediate molecules which act as oxidants toward other wine compounds, as observed in the Fenton reaction. In this case, metal ions such as iron, copper, and manganese are known to be key intermediates in wine oxidation.^12,13^ Surprisingly, the influence of metals on ester hydrolysis has been poorly documented. Patrianakou and Roussis^10^ found that the addition of 0.14 mM (about 7 mg/L) of Fe­(II) favored ester hydrolysis in a Chardonnay wine exposed to air. More recently, Radovanović Vukajlović et al.^11^ reported that Fe, Cu, and Mn, individually and in mixture, could also promote ester hydrolysis, particularly for HAA. Both studies involved exogenous additions of metal ions in finished wines at high but realistic concentrations according to the levels of metal ions reported in wine. Nonetheless, the influence of metal ions naturally present in commercial wines on ester evolution is still unclear. Therefore, this work aimed to investigate the relationship between ester evolution during the artificial aging of thirty-two young commercial white wines and their composition in metal ions and other chemical parameters with the help of advanced statistical tools.

## 2 Materials and Methods

### 2.1 Chemicals

The following ester standards were used to identify and quantify esters: ethyl propanoate (EthC3), ethyl valerate (EthC5), ethyl dodecanoate (EthC12), ethyl-2-methyl butyrate (Eth2mC4), butyl acetate (C4Ac), ethyl phenyl acetate (EPAc), and propyl acetate (C3Ac) were supplied by Sigma-Aldrich Chemie GmbH, (Steinheim, Germany) with purities ≥ 97%. Ethyl butyrate (EthC4), ethyl hexanoate (EthC6), ethyl octanoate (EthC8), ethyl decanoate (EthC10), ethyl isovalerate (EthIC5), hexyl acetate (C6Ac), isoamyl acetate (IsoAc), and octyl acetate (C8Ac) at 99% acquired from Acros Organics (BV, Geel, Belgium). Ethyl isobutyrate (EthIC4) 99% was obtained from Alfa Aesar, and ethyl leucate (EthLeu) 98% was provided by TCI (Tokio chemical industry, Tokio, Japan)­internal standards, ethyl butyrate-4,4,4-d_3_, ethyl hexanoate-d_11_, and ethyl octanoate-d_15_, purchased from C/D/N Isotopes Inc. (Pointe-Claire, QC, Canada) and ethyl trans-cinnamate-d_5_ was supplied by EPTES Sarl (Vevey, Switzerland). In addition, sodium chloride (99%) used for HS-SPME was supplied by Chemlab (Zedelgem, Belgium).

### 2.2 Wine Samples

Thirty-two commercial wines from vintage 2021 were selected from different European countries: Austria (1), Bulgaria (5), Croatia (7), Czech Republic (4), Hungary (4), Romania (2), Serbia (4), Slovakia (2), and Slovenia (3). At the time of analysis, all wines were one-year old and originated from two grape varieties: Chardonnay (13) and Welschriesling (18). Wines were aliquoted in triplicate into 20 mL SPME vials, filled to the brim to minimize headspace, and subjected to forced aging at 30 °C in an oven for 8 weeks. Temperature and time were selected as the optimum conditions to appreciate changes in ester quantities.^11^ Additionally, volatile compounds were quantified before and after temperature treatment.

### 2.3 Enological Parameters and Metal Ion Quantification

Basic parameters, such as ethanol content (% EtOH), total acidity (TA), volatile acidity (VA), and pH, were determined using a WineScanTM analyzer (FOSS, Hillerød, Denmark), a routinely validated FTIR-based platform for wine analysis with integrated calibration and spectral quality-control procedures. Free and total SO_2_ (FSO_2_ and TSO_2_, respectively) were determined using the Ripper method. Metal ion quantification was performed using an Agilent Technologies 7900 ICP-MS instrument, equipped with standard sample introduction components (MicroMist nebulizer, Peltier-cooled Scott-type spray chamber). This setup was used for trace metals, specifically chromium (Cr), manganese (Mn), iron (Fe), cobalt (Co), nickel (Ni), copper (Cu), and zinc (Zn). For elements present in higher concentrations, such as sodium (Na), magnesium (Mg), potassium (K), and calcium (Ca) a Varian 715-ES radial ICP-OES was employed. The methods were performed according to the protocol previously validated with minor adjustments.^14^ To mitigate matrix effects, samples were diluted 10-fold for ICP-MS. The quantification was based on a 10-point external calibration curve ranging from 1 to 1000 μg/L per metal ion.

For ICP-OES analysis, the calibration curves ranged from 0.1 to 40 mg/L. Depending on the element concentration, samples were diluted 2-, 10-, 15-, or 50-fold. Both calibration standards and blanks were prepared in a model wine solution (12% ethanol, 4 g/L glucose, and 4 g/L tartaric acid, adjusted to pH 3.4). All reagents were of an analytical grade or higher. Ultrapure water (Milli-Q, Millipore) and ultrapure nitric acid (HNO_3_, Merck-Suprapure) were used for sample dilution and calibration standard preparation. These were prepared by diluting certified, traceable ICP-grade single-element standards (Merck CertiPUR). Although both analytical methods allowed the detection of additional elements, only these metals were included in the study because they were consistently present across all wine samples independent of their concentration.

Iron speciation (Fe­(II) and Fe­(III)) was determined spectrophotometrically according to the method described by Nguyen and Waterhouse.^15^ Measurements were conducted using a PerkinElmer Lambda 25 UV–Vis spectrophotometer with a 1 nm spectral resolution, recording absorbance at 562 nm. To determine Fe­(II), 1 mL of white wine was added to a cuvette containing 10 μL of ferrozine solution, followed by 1.5 mL of EDTA; absorbance was then measured 1 min after EDTA addition. Total iron was determined separately using the same procedure, but replacing EDTA with 1.5 mL of ascorbic acid to reduce Fe­(III) to Fe­(II). Absorbance for total iron was recorded after approximately 30 min, once the reduction process had stabilized.

### 2.4 Quantification of Esters

Samples were prepared by combining 2 g of NaCl, 3 mL of wine sample, 3 mL of deionized water (Milli-Q water obtained from ELGA PURELAB Option water system supplied by Elga-Veolia, Buckinghamshire, U.K.), and 20 μL of the internal standard (ethyl butyrate-4,4,4d_3_, ethyl hexanoate d_5_, ethyl octanoate d_15_, and ethyl trans-cinnamate d_5_). Volatile compounds were extracted by headspace Solid-Phase Micro-Extraction (HS–SPME) using an SPME fiber assembly (50/30 μm DVB/CAR/PDMS, Stableflex, 24 Ga, Autosampler, Gray (Supelco, St. Louis, MO, USA)). The fiber was conditioned in the SPME Arrow-Conditioning Module for 2 min at 270 °C before being inserted into the headspace of the sample vial for 30 min at 40 °C with simultaneous sample agitation at 250 rpm using a vortex module. It was then transferred to the injector for desorption at 250 °C for 15 min, with a sample injection time of 30 s into the GC column in a GC Agilent 8890 with an MS detector 5977B (Agilent Technologies USA). The separation and detection method was based on that described by Antalick et al.,^16^ with minor adjustments according to a previously published protocol.^17^ The oven temperature was programmed at 40 °C for 5 min, then raised to 200 °C at 3 °C/min, to 240 °C at 10 °C/min, and then held at 240 °C for 10 min. All analyzed compounds were identified by comparing their retention times and mass spectra information with those of pure standards using the NIST Mass Spectral Search Program library for reference. Quantification was performed using Mass Hunter Workstation Software (Version 10.1) from Agilent Technologies. Table S1 presents the calibration curves and other analytical parameters used for the quantification of selected compounds by GC-MS.

Seventeen esters were quantified in all commercial wines before and after forced aging. Before aging, each wine was analyzed once from a single freshly opened bottle. After aging, wines were prepared in triplicate as independent aging replicates, and each replicate was analyzed by a single injection. Multiple injections per replicate were not considered necessary, as the analytical precision and robustness of the method (Table S1) had been previously validated and showed negligible instrumental variability under the analytical conditions used.

The initial concentrations of esters were variable from sample to sample. To summarize the results, esters were grouped as follows according to their sensitivity to hydrolysis: thyl Ester Short-Chain carbon of Fatty Acids (SCEEFA) corresponds to EthC3, EthC4, and EthC5; Ethyl Ester Medium-Chain carbon of Fatty cids (MCEEFA) corresponds to EthC6, EthC8, EthC10, and EthC12. Additionally, Higher Alcohol Acetates (HAA) are composed of C3Ac, C4Ac, C6Ac, and C8Ac. IsoAc was excluded from this group and analyzed separately because of the high initial concentration values compared with other HAAs. Finally, EthIC4, Eth2mC4, EthIC5, EPAc, and EthLeu are in the group of Ethyl Esters of Branched Acids (EEAB).

### 2.5 Statistical Analysis

To explore the sample variability, two different heatmaps were generated, and 34 continuous variables were used, including physicochemical parameters, metal content, and esters (expressed as ratios (*R*): after aging (AA)/before aging (BA) ester concentration), associated with 32 various wine samples from different countries of two varieties, Welschriesling (Welsc.) and Chardonnay (Chard.). By mapping the relationship between wine samples (rows) and variables (columns), the heatmap reveals groups of correlated samples and variables. To create the heatmap, the data were first standardized to have a mean of 0 and a standard deviation of 1. Then, each row of the standardized data was normalized to be between 0 and 100. The data were first standardized by samples (rows), i.e., normalizing each sample independently. This approach enabled the identification of patterns based on how each sample varied across multiple variables. It is particularly suitable for comparing the relative proportions of variables within the individual samples. Moreover, the data were standardized by variables (columns), that is, normalizing each variable independently. This approach helped detect how each variable contributes to the overall patterns in the data. The hierarchical clustering was performed on the sample data using the Euclidean distance between the rows of the standardized data and the default complete linkage method. This method helped visualize the relative importance of each variable in determining the overall patterns.

To further analyze the relationships between 5 groups of ester compounds, expressed as ratios, and 14 variables (metals plus pH, FSO_2_, TSO_2_, and an additional parameter, the proportion of Fe­(II)/Fe­(III)), partial least-squares (PLS) regression was employed. PLS is a multivariate statistical analysis used for both regression and clustering tasks. To set up our PLS-R, we first distinguished between the response variables (*y*-variables) and the predictor variables (*x*-variables). We then used PLS-R on the standardized data to model multiple response variables (*Y*-block) based on multiple predictor variables (*X*-block) simultaneously. The *Y*-block in our PLS-R consisted of the 5 ester families: SCEEFA, MCEEFA, HAA, EEBA, and IsoAc. The *X*-block comprised the remaining 14 input variables. Therefore, the main purpose of using PLS-R in this study was to achieve two objectives: (1) identify the underlying factors that drive variation in the data and (2) correlate and discover the contributions of 14 predictors and the 5 groups of ester compounds in 32 wine samples.

Multicollinearity among predictor variables was not assessed as a separate preprocessing step. This is because PLS-R is specifically designed to handle highly collinear and high-dimensional data by projecting the original predictors onto a set of orthogonal latent variables that maximize covariance with the response variables. Consequently, PLS-R mitigates the instability in coefficient estimation typically associated with multicollinearity, making it particularly suitable for data sets where predictors are expected to be correlated. Moreover, the inspection of the correlation matrix indicates the presence of moderate to strong correlations among several predictor variables, suggesting potential multicollinearity. This further justifies the use of PLS-R, which is specifically designed to handle such data structures.

All computational tasks and multivariate data analyses were performed utilizing data analysis add-on tools in Microsoft Excel, RStudio (R version 4.3.3), IBM SPSS Statistics 25, Minitab 21, SIMCA 14.1, and XLSTAT 2022. The analyses were conducted on a Lenovo PC equipped with an Intel­(R) Core­(TM) i7-1355U CPU @ 1700 MHz and 32 GB of RAM, running the Microsoft Windows 10 OS.

## 3 Results and Discussion

### 3.1 Sample Chemical Characterization

The significant variability in volatile ester concentrations and metal ion levels across commercial wines of the same vintage provided diverse matrices to investigate potential interactions between metal ions and the evolution of these volatile compounds in commercial wines under the same storage conditions. The measurements of basic parameters were within the legal limits of the OIV resolutions for commercial wines. Ethanol levels were measured from 10.8 to 14.3% v/v, total acidity (TA) from 5.1 to 7.9 g/L of tartaric acid, while volatile acidity from 0.2 to 0.5 g/L as acetic acid equivalent, pH ranged from 3.1 to 3.6, total SO_2_ from 50 to 170 mg/L, and free SO_2_ from 7 to 51 g/L. Detailed results for each sample are shown in Table S2. In terms of metal ions, Cr and Co had the lowest concentrations with a maximum of 23 and 9 μg/L, respectively. Ni concentration was measured from 8 to 51 μg/L, Cu ranged from 1 to 182 μg/L, Mn from 310 to 2593 μg/L, and Fe reached levels from 193 to 3204 μg/L. In addition, K, Ca, and Na were measured with concentrations from 328 to 985, 51 to 120, and 7 to 71 mg/L, respectively, and finally Mg from 50 to 90 mg/L. Despite the big standard deviation of the population, all of the concentration values were within the normal range of reported metals in wine around the world.^18,19^

### 3.2 Wine Ester Composition before and after Aging

Table 1 presents the summary values of concentrations before and after aging for each group of esters per wine. MCEEFA stands out as the most representative ester family in terms of concentration before aging, with values ranging from 3248 to 6334 μg/L. Within this family, ethyl hexanoate and ethyl octanoate were the most abundant compounds. IsoAc ranks second in concentration, with values from 720 to 7488 μg/L, and the rest of HAA sum in total up to 300 μg/L. In contrast, the EEBA exhibits much lower concentrations, around 50–150 μg/L approximately. These values overall matched those reported for young white wine.^2,4^ In general, the wine population presents high concentrations of esters, one factor that impacts the hydrolysis of this family of compounds. A high initial concentration of esters generally supposes a faster decrease of them.^20^ Despite our samples presenting a high concentration of esters, it was not possible to generalize their behavior in their evolution during aging across all wines. An important variability between samples was observed irrespective of the initial ester concentrations. The evolution of EEFA and HAA displayed a diminution trend denoting hydrolysis and an increase of EEBA after aging due to esterification as reported in different publications,^2,4^ with some exceptions. On the other hand, esterification of EEBA was significant, especially for EthIC4, a result that agreed with Ling et al.,^21^ who reported that the formation of this compound during aging is faster due to a higher value of the rate constant of formation compared to other branched esters.

**Table 1:** Summary of Quantification in μg/L of Esters before Aging (BA) and after Aging (AA) per Group of Compounds

	SCEEFA		MCEEFA		HAA		IsoAc		EEBA	
family of esters^a^	BA	AA	BA	AA	BA	AA	BA	AA	BA	AA
min	392.0	216.2	3248.8	2680.3	66.9	16.4	720.5	170.8	53.2	49.6
max	675.4	656.1	6334.9	6284.2	320.1	265.2	7488.2	4391.6	177.7	325.4
average	510.4	404.2	4614.7	4122.1	175.2	123.1	2557.8	1294.5	110.8	160.5
median	496.9	391.0	4618.4	4110.2	165.1	116.7	2095.9	1088.8	111.0	152.7
SD^b^	82.1	94.9	797.6	727.2	70.0	68.9	1465.5	876.5	28.7	67.0

a Esters included per family: SCEEFA (EthC3, EhtC4, EthC5); MCEEFA (EthC6, EthC8, EthC10, EthC12); HAAs (C3Ac, C4Ac, C6Ac, C8Ac); EEBA (EthIC4, EthIC5, Eth2mC4, EPAc, EthLeu), and IsoAc (isoamyl acetate) excluded from the groups due to its high concentration.

b SD: standard deviation calculated per group of esters.

Figure 1 illustrates the evolution of ethyl octanoate and isoamyl acetate during artificial aging across samples, in relation to their initial concentrations. These compounds were selected because they are among the most representative esters, in terms of concentration, within the two ester families investigated in this study that are particularly susceptible to hydrolysis during aging, namely, EEFA and HAA. No consistent patterns in the behavior of these compounds were observed. Some wines with lower initial ester concentrations exhibited a higher level of hydrolysis, expressed as the percentage relative difference between concentrations before and after aging ((after aging concentration – before aging concentration)/after aging concentration × 100), compared with wines presenting higher initial concentrations. Moreover, identical initial ester concentrations did not necessarily result in comparable losses during storage, indicating that factors other than postfermentative concentration influence degradation.

**Figure 1:**
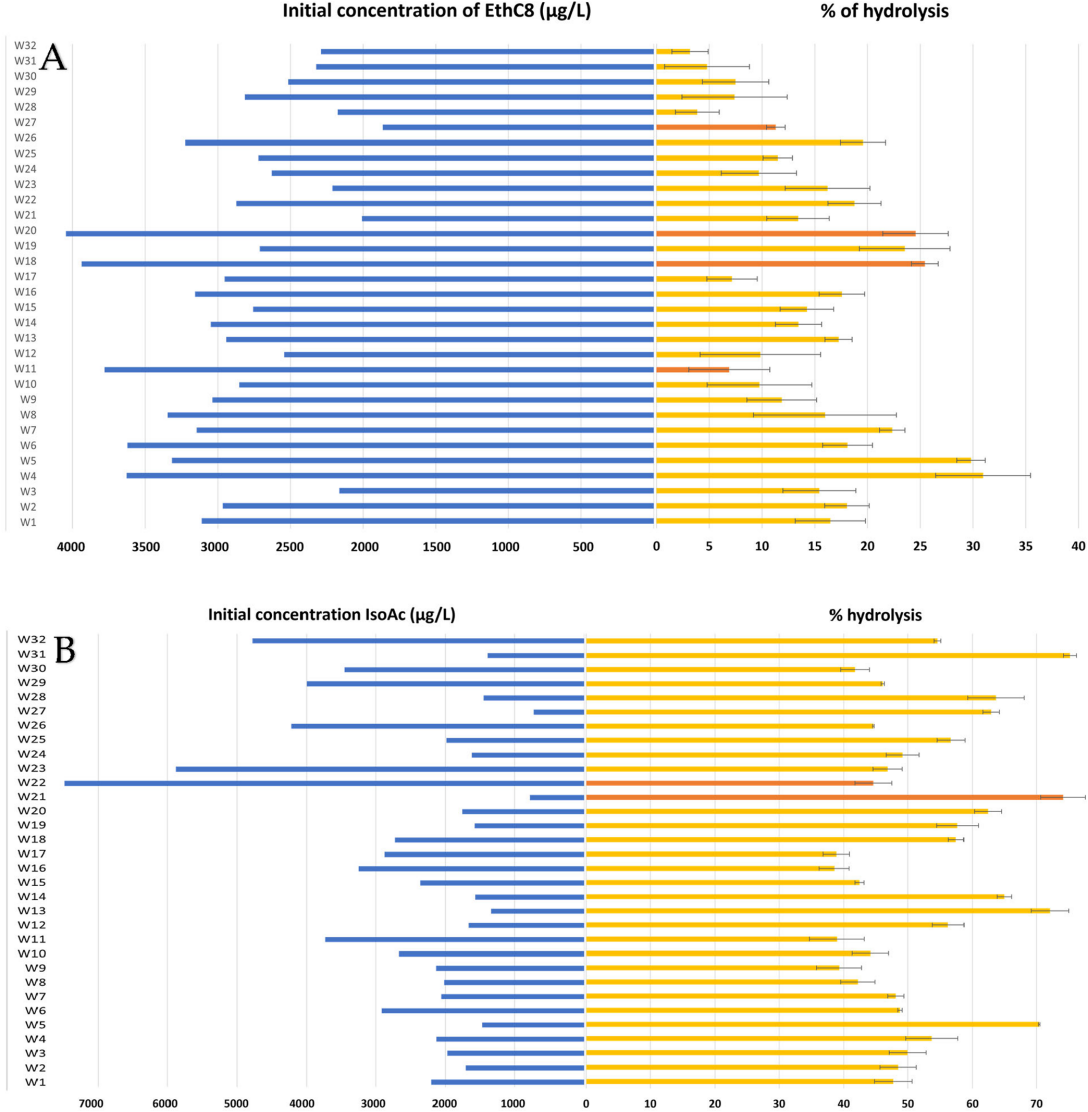
Comparison between the initial concentration of esters. (A) Ethyl octanoate and (B) isoamyl acetate, expressed in μg/L and their percentage of hydrolysis per wine. Error bars represent the standard deviation calculated with the concentrations after aging by triplicate. Colored bars highlight the wine samples discussed in the text as representative examples of nonsystematic hydrolysis behavior.

The degradation behavior also differed between ester classes, with Higher Alcohol Acetates (HAA) exhibiting more pronounced variations than Ethyl Esters of Fatty Acids (EEFA), consistent with previous reports.^2^ This difference is partly due to the concentration differences of the corresponding alcohols between EEFA and HAA. Indeed, ethanol is present in wine at levels 1000-fold higher than higher alcohols making EEFA/ethanol balance closer to equilibrium than for HAA and their corresponding higher alcohols. Nevertheless, the “Law of Mass Action” may not be completely valid to explain hydrolysis in a real wine matrix. In theory, a higher initial concentration of esters should be associated with a higher percentage of hydrolysis; however, this trend was not observed in the sample set. Samples highlighted in orange in Figure 1 exemplify this unexpected behavior, exhibiting hydrolysis percentages that were not proportional to their initial ester concentrations.

Samples w11, w18, and w20 in Figure 1A showed the highest concentration of EthC8 with values between 3500 and 4000 μg/L, whereas sample w27 exhibited a lower concentration of 1865 μg/L. If the initial concentration of ester is the primary factor driving hydrolysis, a greater percentage of degradation would be expected in w11, w18, and w20 compared with w27, but this was not the case. A similar pattern was observed for other esters, where hydrolysis percentages were independent of their initial concentrations. In the second ester example (Figure 1B), wines 21 and 22 are compared to illustrate the absence of a direct relationship between initial concentration and hydrolysis degree. Wine 22 exhibited the highest initial concentration of isoamyl acetate (IsoAc); however, it did not show the highest hydrolysis percentage. Conversely, wine 21 displayed a substantially lower initial concentration but a greater degree of hydrolysis. The concentration of HAA esters varied more among the samples, and their hydrolysis rate was also not predictable.

To better understand this behavior, it was necessary to determine whether a mathematical relationship existed between the initial concentration and its evolution over time. Figure 2 shows the different types of correlation between the initial concentration of esters and their hydrolysis. The graphic shows the relationship between Before Aging (BA) values and Ratios (*R*) for each ester using scatter plots, Spearman correlation coefficient (denoted by “Corr”), coefficient of determination (*R* ^2^), simple linear regression, quadratic regression, and cubic regression. If BA and *R* are linearly related, the data points will align with the linear (red) trendline. In contrast, the presence of a nonlinear relationship will be better described by quadratic (green) or cubic (purple) models. The gray area around the regression lines in the scatter plots represents the confidence interval (significance level α = 0.05). An *R* ^2^ = 1 showing a perfect fit (100% of the variance in *R* is explained by BA).

**Figure 2:**
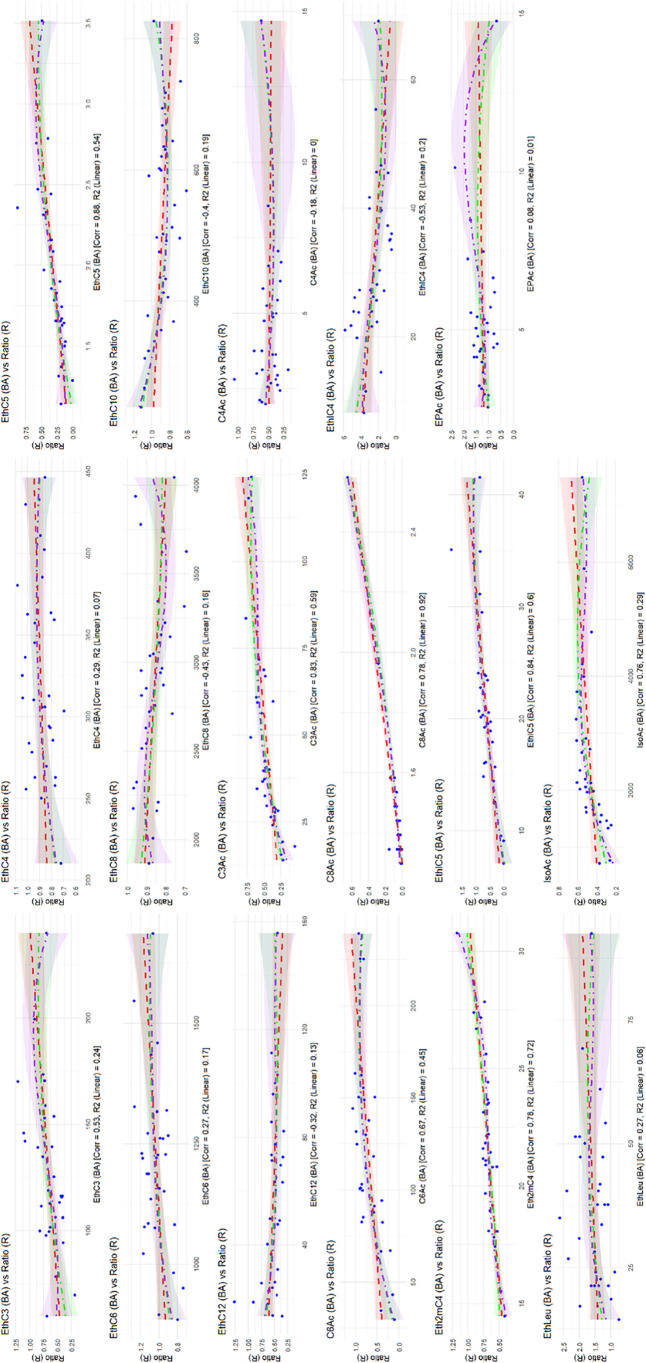
Relationship between the before aging (BA) concentration (μg/L) and the ratios (*R*) using scatter plots. Spearman correlation coefficient (denoted by “Corr”), coefficient of determination (*R* ^2^), simple linear regression (red), quadratic regression (green), and cubic regression (purple).

According to this wine data set, only some compounds showed a clear relationship, not necessarily linear, between the initial concentration and the ratio of hydrolysis. In Figure 2, EthC5, C3Ac, C8Ac, Eth2mC4, EthIC5, and IsoAc exhibited correlation values higher than 0.7. Acetates were a group of compounds which presented a strong relationship between their initial concentration and ratios. Negative correlations were expected, which would mean that hydrolysis is higher (smaller *R*) when the initial concentration is higher. However, those compounds that presented any impact had positive values (even isoamyl acetate: Corr: 0.76, IsoAc). These results suggest that the evolution of acetates during artificial aging might have reflected the wine matrix effect on their hydrolysis occurring already in the bottle and in the cellar before. In other words, wines with higher initial concentrations might have been partly connected to better ester preservation before the experiment, and this effect might have lasted during the artificial aging. In the case of EEFA, their hydrolysis could not be linked to their initial contents in wines. Some compounds of EEBA exhibited moderate correlation; however, Eth2mC4 showed the highest correlation, with a coefficient of 0.78.

These results indicate that, although only two grape varieties were included in this study, the wines exhibited substantial compositional diversity, effectively representing 32 distinct matrices. In this context, the evolution of postfermentation esters during aging suggests that hydrolysis cannot be attributed to a single matrix-related factor. Rather, multiple matrix components appear to act in combination to modulate the magnitude of ester variation, with potential consequences for the sensory profile of the wines. The wine matrix composition is not that simple, and several compounds can interact with each other, especially over time. Many articles have reported that different features can disrupt the ester’s evolution during storage. Apart from the initial ester concentration, pH, SO_2_, antioxidants, polyphenols, and metal ions have been reported to influence hydrolysis, acting as boosters or inhibitors.^6,11,21,22^

### 3.3 Patterns of Variability in White Wine Matrices

For the data processing, the concentrations of selected metals (Na, Mg, K, Ca, Cr, Mn, Fe, Co, Ni, Cu, Zn), along with fundamental enological parameters such as ethanol content, pH, volatile acidity, total acidity, and free and total SO_2_, were included as independent variables. The objective was to assess their potential association with observed ester ratios. The heatmaps (Figure 3) correspond to a total of 34 continuous variables. The variability of the content of all of the parameters evaluated per sample can be appreciated according to the change in the darkness and shown in the heatmaps.

**Figure 3:**
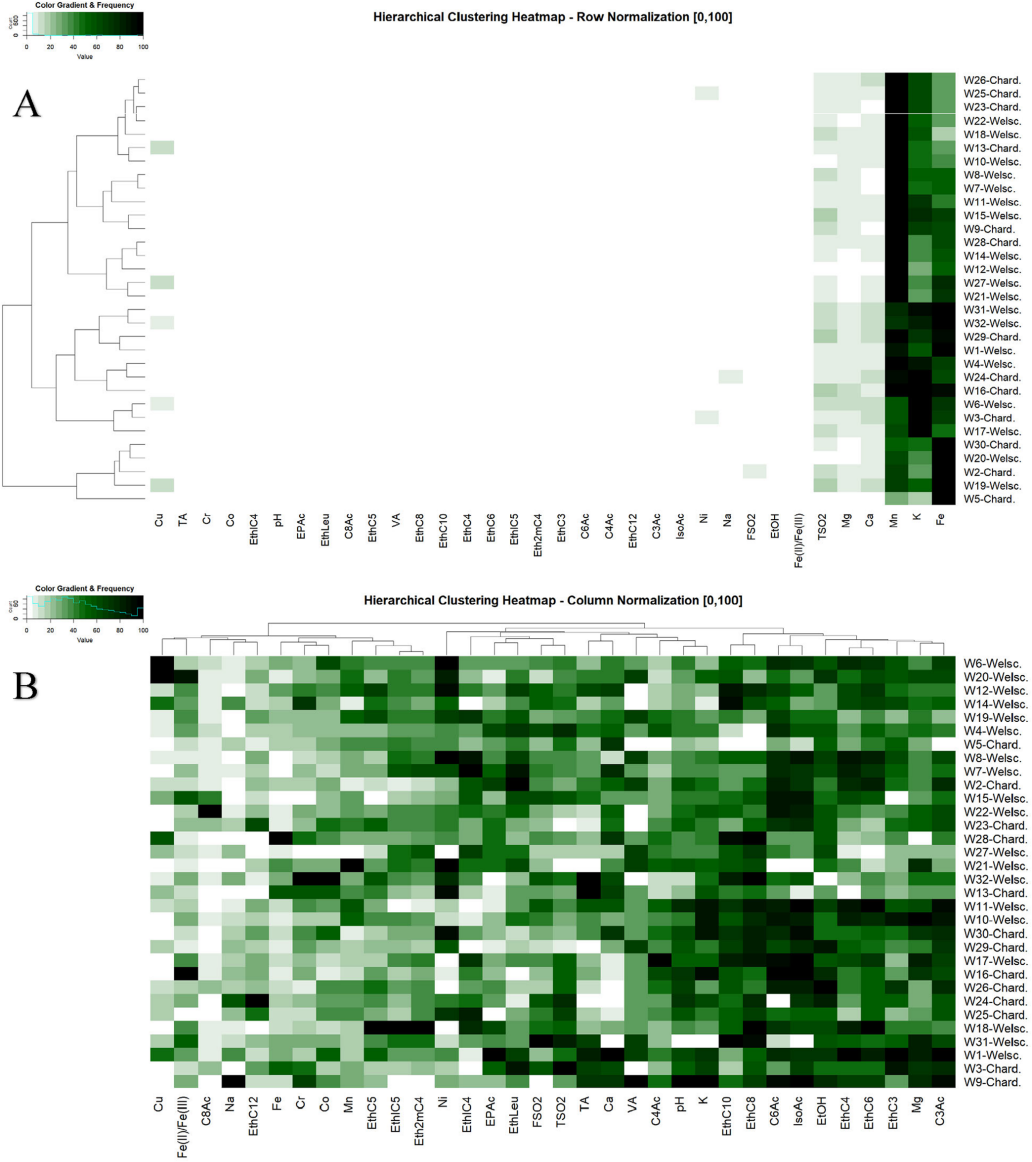
Hierarchical clustering heatmap of the content of 34 variables (basic parameters, metals and esters’ ratio before forced aging/after forced aging concentrations) with two types of normalizations: by samples (A) and by variables (B).

When the heatmap was normalized by rows (samples; Figure 3A), the clustering reflected similarities in the relative composition of the wines, leading to the identification of seven distinct sample groups. Fe, K, and Mn emerged as the main variables driving this classification, followed by Ca and Mg, highlighting the dominant role of metals in shaping the compositional profiles of the wines. In contrast, ester ratios appeared with low relative intensity and without a consistent pattern across samples, indicating a high intersample variability in ester behavior during aging. Consequently, no specific ester family was able to drive sample grouping or classification under the row-normalized conditions. In contrast, when the heatmap was normalized by columns (variables; Figure 3B), the differences in the relative distribution of individual variables across the samples are highlighted, including the ratio of esters. This approach indicates that differences among samples are driven by specific variable–sample interactions rather than by a compositional profile.

Although this approach did not reveal a clear separation of samples or any classification related to the variety or origin, it emphasized the wide diversity of parameter combinations present in the data set.

### 3.4 Exploring the Influence of Wine Matrix on Ester Evolution

To further analyze the relationships between ester compounds and other 14 variables (metals, including the proportion of Fe­(II)/Fe­(III), pH, FSO_2_, and TSO_2_) across 32 wine samples, partial least-squares regression (PLS-R) was applied, a multivariate statistical analysis used for both regression and clustering tasks. In this model, the *Y*-block in our PLS-R consisted of the grouped esters in 5 families (SCEEFA, MCEEFA, HAA, IsoAc, and EEBA); however, the *Y*-block was constructed using the recalculated evolution ratios (*R*) of each ester family, and the *X*-block comprised the other 14 predictor variables: metals (Na, Mg, K, Ca, Cr, Mn, Fe, Co, Ni, Cu, Zn) along with pH, FSO_2_, and TSO_2_.

Therefore, the main purpose of using PLS-R in this study was to achieve two objectives: (1) identify the underlying factors that drive variation in the data and (2) correlate and discover the contributions of 14 predictors and the 5 groups of ester compounds in 32 wines. The PLS-R model demonstrated how selected factors contribute to the measured concentration variations in the ester groups expressed as the ratio *R*, defined earlier.

Table S3 summarizes the results of the analysis, which shows that our PLS-R model with |LV| ≥ 8 can explain at least 80% of the variation in the *X*-block data (*R* ^2^ *X* ≥ 80%) and at least 55% of the variation in the *Y*-block data (*R* ^2^ *Y* ≥ 55%). Choosing |LV| = 10 (i.e., most metals) can even better explain the variation in the *X*-block and *Y*-block data by approximately 91 and 57%, respectively, with an average model Goodness-of-Fit of 57%. In other words, 57% of the ester variations could be explained by the 10 main variables of *X*-block data. This indicates that there are still more inherent factors that need to be taken into account when investigating the wine matrix effect on ester variations during aging.

On the basis of the response variables (esters) in the PLS-R loading plots and the graphical representation of the associations of variables on the LVs depicted in Figure 4 (i.e., the PLS loadings), the behavior of the esters is differentiated and impacted by different parameters. The plot represents two latent variables (*t*_1_ and *t*_2_). *t*_1_ is characterized by strong positive correlations with K, pH, IsoAc, HAA, Mg, and Na, and by a low-moderated positive correlation with MCEEFA, SCEEFA, and Fe­(II)/Fe­(III). On the other hand, *t*_2_ is strongly associated with Cr, Co, Ca (positive correlation), and EEBA (negative correlation). This confirms that the behavior of EEBA is different from that of other types of esters. From an enological perspective, this group of esters increased in concentration during aging, whereas other ester groups showed a decreasing trend.^2,4,5^ By contrast, Fe showed a correlation with both variables *t*_1_ and *t*_2_, suggesting a complex role of iron in ester hydrolysis with a negative correlation at *t*_1_ and a positive correlation at *t*_2_.

**Figure 4:**
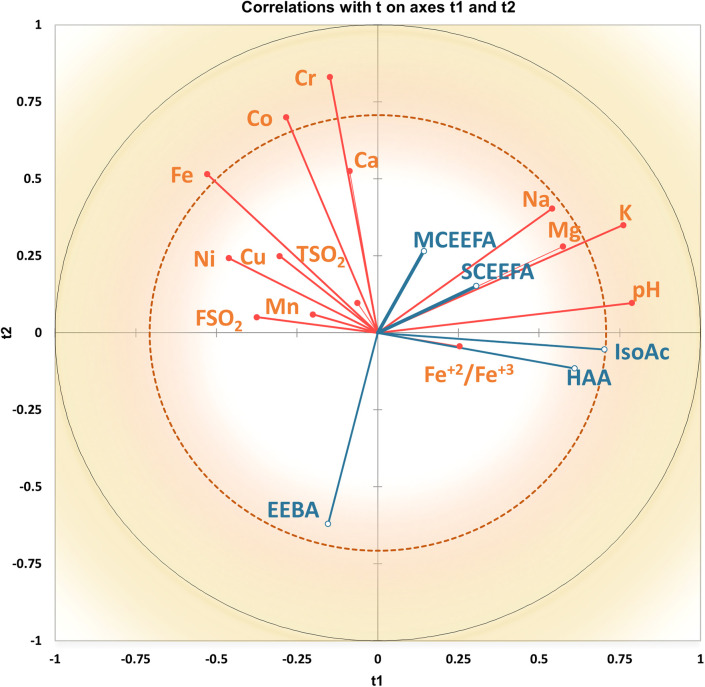
PLS-R loading plots (correlation circle plot) of all parameters (metal ions plus pH, FSO_2_, and TSO_2_) and ester families, SCEEFA, MCEEFA, HAA, IsoAc, and EEBA (*p* < 0.05), on the first two latent components.

Nevertheless, this statistical approach showed that less than 60% of the phenomena can be explained with all of these parameters (14 predictors), including pH. Within this set of wines, pH appears to be an important determinant of ester behavior, particularly for Higher Alcohol Acetates, including isoamyl acetate. Consistent with Marais,^6^ who reported a stronger effect of pH on HAA degradation than on EEFA, the present study also showed a strong association between pH and HAA, whereas the correlation with EEFA was comparatively weak. This also means that other important factors besides pH are strongly involved in ester hydrolysis. Additionally, HAA ratios were also well positively correlated with K, Na, and Mg. They can affect the pH values of wine, increasing the value^23,24^ and then could act as potential inhibitors of ester hydrolysis in an indirect sense, meaning that pH is the main factor for hydrolysis. The correlation of K with pH is already evident, and it has been reported.^23^ However, the fact that Na and Mg can influence the hydrolysis has not been established; their presence seems to indirectly influence the inhibition of the ester hydrolysis of HAAs by modifying the physicochemical properties of the wine matrix.

In the case of Cu, Mn, and Fe, which have previously been reported to influence the hydrolysis of long-chain ethyl esters of fatty acids,^11^ only Fe showed a moderate negative correlation with HAA ratios, suggesting that Fe has an impact on the hydrolysis of this group of esters. While Fe is well-known for catalyzing oxidative reactions in wine,^12,25^ these results indicate that Fe may also play a role in modulating ester hydrolysis, as it was shown in previous studies.^10,11^ On the other hand, despite Cu and Mn being identified as influential variables (Figure 5), they did not exhibit a clear correlation with esters in this data set, meaning that those metals did not contribute substantially to the model. This result contrasts with a recent study suggesting that an exogenous addition of Cu and Mn could promote ester hydrolysis.^11^ Nevertheless, metal concentrations in the present study were measured at lower levels than those of the additions made in the previous work. It also indicates that interactions between metal ions and esters might differ between the source of metal, exogenous, or natural presence. Metal availability may differ depending on whether the metals are naturally present within the wine matrix or added after vinification once the product is already finished. Metals already present in wine can interact with and become complexed with matrix constituents, potentially limiting their participation in further chemical reactions. Interestingly, Fe showed more consistency with previous work and seems to consistently favor ester hydrolysis, particularly in the case of Higher Alcohol Acetates. Additionally, Co, Cr, and Ca may have an impact on the esterification of the EEBA. None of these parameters showed a direct impact on the hydrolysis of the EEFA.

**Figure 5:**
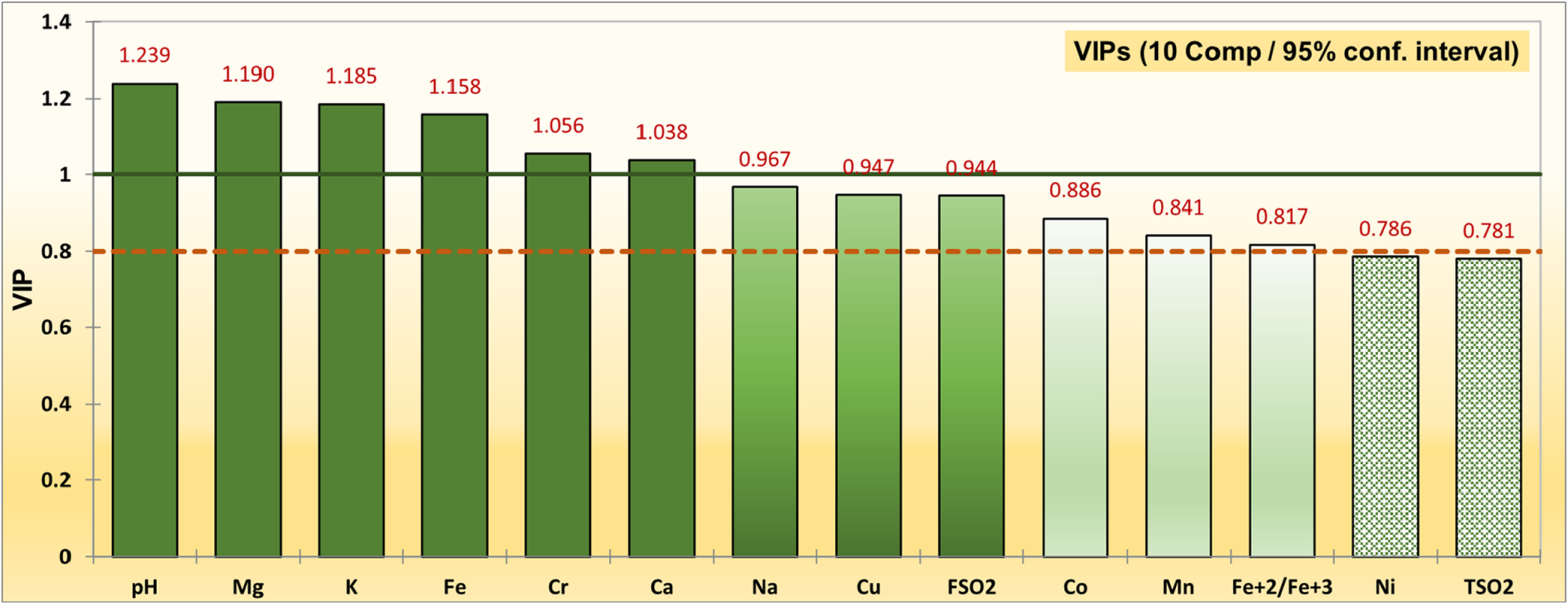
VIP scores associated with the predictors from PLS-R (with 10 LVs) performed for the 32 samples and 14 parameters (metal ions plus pH, FSO_2_, and TSO_2_).

Conversely, *t*_3_ (Table S4) reports robust positive correlations with FSO_2_, TSO_2_ and a moderate correlation with Fe­(II)/Fe­(III), indicating that this component represents the factors related to the oxidative environment. The moderate correlations of SCEEFA and MCEEFA with *t*_3_ suggest these variables might also be influenced by factors related to sulfur dioxide presence and the ratio of iron. The action of Fe in oxidative conditions in wine has been defined as a cycle from Fe­(II) to Fe­(III), able to boost a chain of reactions to form highly reactive free radicals.^12^ Polyphenols are the major target for hydroperoxyl radicals, and SO_2_ plays the role of protection against wine oxidation, where quinones can return as hydroquinone form, but also they can “neutralize” those hydroperoxyl radicals.^26,27^ SO_2_ has been reported as protective for esters during aging for ethyl esters of fatty acids, especially for ethyl octanoate and ethyl decanoate,^22^ matching other results suggesting a boosting effect of O_2_ presence toward the hydrolysis of those specific esters.^10,11^ However, the mechanisms of how this protection is done for esters have not been elucidated yet and have not been related to metals either.

The PLS-R model with 10 LVs identified 9 predictor variables with VIP scores ≥ 0.9, including pH, Mg, K, Fe, Cr, Ca, Na, and Cu. These predictors had the greatest influence on the response variables, either positively or negatively, specifically the groups of esters. The VIP scores plot (Figure 5) associated with the predictors reveals that, besides pH, metals such as K, Mg, and Fe are the primary drivers of ester evolution and other chemical properties in our data set. These variables are strongly aligned with the first latent component (*t*_1_) and have high VIP scores, indicating their significant influence. Other metals, such as Na, Co, and Ni, played a limited role in the explanation of esters’ behavior over time. Matrix interactions are complex, and despite the importance of pH in this model and the addition of other 13 variables, they could not explain more than 57% of the ester variations in the present study.

In summary, this study demonstrates that the esters’ behavior during aging may not be attributed to a single factor. Although pH and ester postfermentative content influence the ester hydrolysis in young white wines, metals also contribute to the modulation of the hydrolysis rate. The use of commercial wines allowed the investigation of ester evolution in the presence of naturally occurring metal compositions, avoiding the exclusive use of artificially spiked model systems, highlighting the possible role of metals on the esters’ behavior. However, metals alone do not fully explain the observed variability, indicating that, beyond the compounds traditionally involved in ester hydrolysis, additional wine matrix components and their interactions likely contribute to the modulation of these reactions over time. Consequently, no single factor can predict the long-term evolution of these compounds in a bottle, as their behavior is strongly matrix-dependent.

## Supplementary Material



## Notes

Calibration and validation parameters for the quantification of selected compounds by GC-MS (Table S1); detailed results of wine basic parameters (Table S2); summary of results of the model quality from the partial least-squares (PLS-R) regression analysis (goodness-of-fit) for each group of ester compounds (Table S3); and correlation matrix between the measured variables and the latent variables (*t*) (Table S4) (PDF)

## Abbreviations

AA: after aging
Al: aluminum
BA: before aging
C3Ac: propyl acetate
C4Ac: butyl acetate
C6Ac: hexyl acetate
C8Ac: octyl acetate
Ca: calcium
Co: cobalt
Corr: Spearman correlation coefficient
Cr: chrome
Cu: copper
Ea: activation energy
EDTA: ethylenediaminetetraacetic acid
EEBA: ethyl ester of branched acids
EEFA: ethyl ester of fatty acids
EPAc: ethyl phenyl acetate
ER: ester ratio
Eth2mC4: ethyl-2-methyl butyrate
EthC10: ethyl decanoate
EthC12: ethyl dodecanoate
EthC3: ethyl propanoate
EthC4: ethyl butyrate
EthC5: ethyl valerate
EthC6: ethyl hexanoate
EthC8: ethyl octanoate
EthIC4: ethyl isobutyrate
EthIC5: ethyl isovalerate
EthLeu: ethyl leucate
EtOH: ethanol
Fe: iron
FSO_2_: free sulfur dioxide
GC-MS: gas chromatography coupled to mass spectrometry
HAA: higher alcohol acetates
HNO_3_: nitric acid
HS-SPME: headspace–solid-phase microextraction
IC4Ac: isobutyl acetate
ICP-MS: inductively coupled plasma–mass spectrometry
ICP-OES: inductively coupled plasma–optical emission spectroscopy
IsoAc: isoamyl acetate
K: potassium
MCEEFA: ethyl ester of medium-chain fatty acids
Mg: magnesium
Mn: manganese
Na: sodium
NaCl: sodium chloride
Ni: nickel
pH: potential of hydrogen
PLS-R: partial least-squares regression
r: reaction rate
R: ester ratio
*R* ^2^: coefficient of determination
SCEEFA: ethyl ester of short-chain fatty acids
Sn: tin
SO_2_: sulfur dioxide
TA: total acidity
TSO_2_: total sulfur dioxide
VA: volatile acidity
VIP: variable importance in projection
Zn: zinc
