# Supplementary material for: Revisiting the Influence of Young White Wine Composition on Ester Evolution
Source: J Agric Food Chem. 2026 Jun 18;74(25):19950–61. doi: 10.1021/acs.jafc.6c04425 (PMC13455054; doi:10.1021/acs.jafc.6c04425)
Supplement: Supplementary file 1 [file jf6c04425_si_001.pdf]

Supporting information

Revisiting the influence of young white wine composition on ester evolution

Diana A. Martin-Rojas ⸙, Tatjana Radovanović Vukajlović ⸙, Martin Šala ‡, Vid Šelih ‡, Valentina Obradović §, Josip Mesić §, Adrian Hermes⌖, Melita Sternad Lemut ⸙, Guillaume Antalick ⸙.*

⸙ *Wine Research Centre, University of Nova Gorica, Lanthieri Palace, Glavni trg 8, 5271 Vipava, Slovenia.*

‡ *National Institute of Chemistry, Hajdrihova 19, 1000, Ljubljana, Slovenia.*

§ *Faculty of Tourism and Rural Development Požega, Josip Juraj Strossmayer University of Osijek, Vukovarska 17, 34000 Požega, Croatia.*

⌖ *University of Nova Gorica, Centre for Information Technologies and Applied Mathematics, Lanthieri Palace, Glavni trg 8, 5271 Vipava, Slovenia.*

Table S1. Calibration and validation parameters for the quantification of selected compounds by GC-MS.

Ester compound	SIM ions (m/z)[i]	Calibration range (μg/L)	Calibration curve	R^2	Recovery[ii] (%)	RSD[iii] (%)	LOD[iv] (μg/L)	LOQ[iv] (μg/L)
**Ethyl propanoate[v]**	**102**/57/75	1 - 250	$y = 0.0002x + 0.010$	0.991	105	8.7	0.3	1.1
**Ethyl isobutyrate[v]**	**116**/88/71	1 - 250	$y = 0.0007x + 0.036$	0.999	127	9.4	0.3	1.1
**Propyl acetate[v]**	**61**/43	2 - 250	$y = 0.0001x + 0.003$	0.996	103	9.5	0.7	2.2
**Ethyl butyrate[v]**	**88/**71/60	0.5 - 250	$y = 0.0021x + 0.072$	0.995	104	8.4	0.2	0.6
**Ethyl 2-methylbutyrate[v]**	**102**/57/85	0.5 - 250	$y = 0.0052x - 0.057$	0.998	101	7.3	0.1	0.4
**Ethyl isovalerate[v]**	**88**/85/57	0.5 - 250	$y = 0.0051x - 0.004$	0.998	94	7.5	0.1	0.2
**Butyl acetate[v]**	**56**/43	0.5 - 250	$y = 0.0002x - 0.001$	0.995	113	21	0.3	0.8
**Isoamyl acetate[v]**	**70**/55/43	10 - 5000	$y = 0.0056x - 0.419$	0.998	100	6.4	0.5	1.7
**Ethyl valerate[v]**	**85**/88/101	0.5 - 50	$y = 0.0070x - 0.006$	0.998	97	6.3	0.1	0.4

[i] Qualifier and quantifier ions (indicated in normal and bold font, respectively) were selected according to the original method validated by Antalick et al. (2010).
[ii] Wines were spiked at 100 μg/L with all esters, except isoamyl acetate (2000 μg/L); ethyl hexanoate and ethyl octanoate (1000 μg/L); hexyl acetate, ethyl leucate and ethyl decanoate (200 μg/L); ethyl valerate, ethyl phenyl acetate and ethyl dodecanoate (20 μg/L).
[iii] Relative standard deviations (RSD) were calculated using eight consecutive injections of the same wine sample.
[iv] LOD and LOQ were calculated according to the signal-to-noise ratios of 3 and 10, respectively.
[v] Ethyl butyrate-4,4,4-d_3

**Ethyl hexanoate**[vi]	**88**/99/60	5 - 2500	*y = 0.0008x + 0.654*	0.997	97	8.7	0.1	0.5
**Hexyl acetate**[vi]	**56**/43	1 - 500	*y = 0.0012x - 0.011*	0.998	88	9.5	0.2	0.6
**Ethyl octanoate**[vii]	**88**/101/127	5 - 2500	*y = 0.0006x + 0.038*	0.995	107	8.0	0.1	0.4
**Octyl acetate**[vii]	**70**/56/43	0.5 -50	*y = 0.0021x - 0.003*	0.999	90	20	0.1	0.3
**Ethyl leucate**[vi]	**87**/76/104	1 - 500	*y = 0.0001x + 0.001*	0.996	98	16	0.3	1.1
**Ethyl decanoate**[vii]	**88**/101	1 - 500	*y = 0.0077x + 0.484*	0.990	93	5.2	0.2	0.7
**Ethyl phenyl acetate**[viii]	**91**/105	0.5 - 50	*y = 0.5367x - 0.252*	0.998	91	15	0.1	0.2
**Ethyl dodecanoate**[vii]	**88**/101	0.5 - 50	*y = 0.0048x - 0.005*	0.999	119	15	0.3	0.8

[vi] Ethyl hexanoate-d_5
[vii] Ethyl octanoate-d_{15}
[viii] Ethyl trans-cinnamate-d_5

Table S2. Detailed measurements of wine's basic parameters: EtOH (Ethanol), TA (Total acidity), VA (Volatile acidity), measured by FOSS; and FSO_2 (Free sulphur dioxide), and TSO_2 (total sulphur dioxide) determined by the Ripper method.

Wine	Variety	Country	EtOH (% v/v)	TA (g /L tartaric acid)	VA (g/L acetic acid)	pH	FSO_2 (g/L)	TSO_2 (g/L)
**1**	Welschriesling	Czech Republic	13.390	7.400	0.400	3.410	17.3 ± 0.37	109 ± 0.30
**2**	Chardonnay	Czech Republic	12.260	6.300	0.400	3.380	23.0 ± 0.37	81.8 ± 0.30
**3**	Chardonnay	Slovakia	12.430	7.000	0.200	3.360	32.4 ± 0.37	170 ± 0.52
**4**	Welschriesling	Slovakia	12.750	6.500	0.300	3.280	36.0 ± 1.28	142 ± 0.52
**5**	Chardonnay	Bulgaria	12.610	5.800	0.200	3.180	16.8 ± 0.74	110 ± 0.80
**6**	Welschriesling	Bulgaria	12.850	5.700	0.300	3.300	23.9 ± 0.64	99.3 ± 0.30
**7**	Welschriesling	Croatia	12.960	6.000	0.300	3.250	26.6 ± 0.37	94.2 ± 0.30
**8**	Welschriesling	Croatia	13.120	6.000	0.400	3.270	24.3 ± 0.64	92.4 ± 0.00
**9**	Chardonnay	Czech Republic	13.500	7.200	0.500	3.620	7.20 ± 0.37	88.1 ± 0.00
**10**	Welschriesling	Croatia	12.560	6.100	0.300	3.300	26.0 ± 0.98	85.0 ± 0.30
**11**	Welschriesling	Croatia	13.220	6.700	0.300	3.490	32.8 ± 0.64	94.8 ± 0.52

**12**	Welschriesling	Romania	11.950	6.900	0.200	3.200	31.1 ± 0.37	103 ± 0.30
**13**	Chardonnay	Romania	12.040	7.800	0.300	3.190	21.8 ± 0.37	84.9 ± 0.30
**14**	Welschriesling	Hungary	11.940	5.600	0.200	3.210	31.2 ± 0.37	110 ± 0.30
**15**	Welschriesling	Austria	12.550	6.500	0.300	3.280	35.5 ± 1.28	121 ± 0.30
**16**	Chardonnay	Hungary	13.530	5.900	0.300	3.450	16.3 ± 0.00	112 ± 1.05
**17**	Welschriesling	Hungary	12.850	5.800	0.300	3.350	25.0 ± 0.37	113 ± 0.30
**18**	Welschriesling	Hungary	12.940	5.800	0.300	3.250	29.2 ± 0.37	110 ± 0.00
**19**	Welschriesling	Croatia	12.460	6.100	0.400	3.290	20.4 ± 0.37	91.6 ± 0.32
**20**	Welschriesling	Croatia	13.270	6.300	0.400	3.240	12.3 ± 0.00	94.7 ± 0.30
**21**	Welschriesling	Croatia	12.440	5.100	0.400	3.370	17.3 ± 0.98	54.4 ± 0.30
**22**	Welschriesling	Slovenia	12.760	5.400	0.200	3.320	19.2 ± 0.00	69.9 ± 0.30
**23**	Chardonnay	Slovenia	12.760	5.300	0.200	3.340	16.6 ± 0.64	50.3 ± 0.30
**24**	Chardonnay	Bulgaria	13.760	5.100	0.300	3.470	33.7 ± 0.37	143 ± 0.80
**25**	Chardonnay	Bulgaria	12.720	5.600	0.300	3.370	26.6 ± 0.37	134 ± 1.05

**26**	Chardonnay	Bulgaria	14.310	5.400	0.300	3.440	14.0 ± 0.00	113 ± 0.52
**27**	Welschriesling	Slovenia	13.030	5.600	0.400	3.270	14.6 ± 0.37	73.2 ± 0.00
**28**	Chardonnay	Serbia	12.830	5.800	0.300	3.260	24.1 ± 0.37	90.0 ± 0.30
**29**	Chardonnay	Serbia	13.810	5.200	0.400	3.390	13.7 ± 0.37	57.6 ± 0.00
**30**	Chardonnay	Serbia	12.410	6.200	0.300	3.410	20.4 ± 0.64	78.5 ± 0.30
**31**	Welschriesling	Serbia	12.830	7.000	0.400	3.050	51.7 ± 0.37	146 ± 0.30
**32**	Welschriesling	Czech Republic	10.830	7.900	0.200	3.130	32.0 ± 0.37	104 ± 0.52
	**Min**		10.830	5.100	0.200	3.050	7.160	50.300
	**Max**		14.310	7.900	0.500	3.620	51.710	170.240
	**Average**		12.802	6.156	0.313	3.316	24.141	100.694
	**Median**		12.795	6.000	0.300	3.300	23.995	97.080
	**SD**		0.650	0.756	0.079	0.115	9.022	27.176

Table S3. Summary of results of the model quality from the partial least-squares (PLS-R) regression analysis (goodness-of-fit) for each group of ester compounds.

Y-BLOCK (I.E.. AROMATIC GROUPS) (RESPONSE VARIABLES. N = 5) ↓	#Latent Variables (i.e.. |LV|)	R-Sq (R^2)	Average Model Goodness-of-Fit (avg. R^2)	Captured Cumulative X-block Variance (R^2X cum)	CAPTURED CUMULATIVE Y-BLOCK VARIANCE (R^2Y CUM)
**SCEEFA**		0.394			
**MCEEFA**		0.268			
**HAAS**	7	0.502	**0.454**	0.852	**0.455**
**ISOAC**		0.621			
**EEBA**		0.485			
**SCEEFA**		0.395			
**MCEEFA**		0.271			
**HAAS**	8	0.523	**0.459**	0.930	**0.460**
**ISOAC**		0.621			
**EEBA**		0.488			
**SCEEFA**		0.395			
**MCEEFA**		0.271			
**HAAS**	9	0.528	**0.463**	0.963	**0.464**
**ISOAC**		0.623			
**EEBA**		0.499			
**SCEEFA**		0.401			
**MCEEFA**		0.272			
**HAAS**	10	0.528	**0.464**	1.000	**0.465**
**ISOAC**		0.623			
**EEBA**		**0.499**			

Table S4. Correlation matrix between the measured variables and the latent variables (t).

Metal	t1	t2	t3	t4	t5	t6	t7	t8	t9	t10
**K**	0.761	0.350	-0.202	0.088	-0.012	0.110	-0.052	-0.220	-0.219	0.052
**Ca**	-0.087	0.525	-0.393	0.463	-0.057	-0.385	0.177	0.092	0.104	0.284
**Na**	0.541	0.404	-0.082	-0.398	0.009	-0.097	0.214	0.189	0.199	-0.382
**Mg**	0.573	0.281	0.372	0.213	0.363	0.176	0.058	-0.201	0.335	0.190
**Cr**	-0.148	0.830	-0.032	-0.309	-0.111	-0.087	-0.111	-0.087	0.207	0.207
**Mn**	-0.201	0.060	-0.258	0.212	0.478	0.382	0.174	-0.539	-0.189	0.155
**Fe**	-0.529	0.515	-0.152	-0.162	0.221	-0.027	-0.024	0.160	-0.357	0.134
**Co**	-0.283	0.699	-0.142	0.116	-0.379	0.072	0.017	-0.183	0.030	-0.139
**Ni**	-0.461	0.243	-0.364	0.305	-0.054	0.193	0.098	-0.446	0.210	-0.329
**Cu**	-0.304	0.250	0.021	0.422	0.185	0.120	-0.345	0.426	-0.167	-0.471
**Fe(II)/Fe(III)**	0.253	-0.044	0.379	0.119	0.308	-0.378	-0.652	-0.213	0.039	-0.165
**pH**	0.787	0.096	-0.305	-0.030	-0.050	0.187	0.174	0.023	-0.384	-0.037
**FSO_2**	-0.376	0.051	0.825	-0.054	-0.193	0.019	0.126	-0.153	-0.068	0.122
**TSO_2**	-0.063	0.097	0.723	-0.108	0.020	-0.414	0.247	-0.035	-0.416	-0.156
**SCEEFA**	0.305	0.152	0.320	0.467	0.085	0.078	0.230	0.175	0.024	0.069
**MCEEFA**	0.143	0.266	0.376	-0.166	-0.212	0.367	-0.121	0.032	0.065	0.012
**HAAs**	0.610	-0.115	0.008	0.311	-0.284	-0.088	-0.196	0.019	0.003	0.158
**IsoAc**	0.703	-0.054	-0.003	0.154	-0.410	0.055	-0.049	-0.065	-0.177	0.049
**EEBA**	-0.154	-0.621	0.006	-0.001	-0.086	-0.140	0.133	-0.191	0.076	-0.090
